# Comprehensive characterization of 11 prognostic alternative splicing events in ovarian cancer interacted with the immune microenvironment

**DOI:** 10.1038/s41598-021-03836-1

**Published:** 2022-01-19

**Authors:** Congbo Yue, Tianyi Zhao, Shoucai Zhang, Yingjie Liu, Guixi Zheng, Yi Zhang

**Affiliations:** 1 Department of Clinical Laboratory, Qilu Hospital of Shandong University, Jinan, Shandong Province 250012 People’s Republic of China; 2 Department of Obstetrics and Gynecology, Qilu Hospital of Shandong University, Jinan, Shandong Province 250012 People’s Republic of China

**Keywords:** Cancer, Computational biology and bioinformatics

## Abstract

Alternative splicing (AS) events play a crucial role in the tumorigenesis and progression of cancer. Transcriptome data and Percent Spliced In (PSI) values of ovarian cancer patients were downloaded from TCGA database and TCGA SpliceSeq. Totally we identified 1472 AS events that were associated with survival of ovarian serous cystadenocarcinoma (OC) and exon skipping (ES) was the most important type. Univariate and multivariate Cox regression analysis were performed to identify survival-associated AS events and developed the prognostic model based on 11-AS events. The immune cells and different response to cytotoxic T lymphocyte associated antigen 4 (CTLA-4) and programmed cell death protein 1 (PD-1) blockers in low-risk and high-risk group of OC patients were analyzed. Ten kinds of immune cells were found up-regulated in low-risk group. Activated B cell, natural killer T cell, natural killer cell and regulatory T cell were associated with survival of OC. The patients in low-risk group had good response to CTLA-4 and PD-1 blockers treatment. Moreover, a regulatory network was established according to the correlation between AS events and splicing factors (SFs). The present study provided valuable insights into the underlying mechanisms of OC. AS events that were correlated with the immune system might be potential therapeutic targets.

## Introduction

Ovarian cancer is the leading cause of gynecologic cancer-related death in developed countries, and it ranks the sixth most prevalent form of cancer worldwide. Nearly 70% of ovarian cancer patients are diagnosed at an advanced stage, and the 5-year survival rate is below 30%^1,2^. It is generally believed that dysregulation of gene expression plays an important role in the occurrence and development of tumors. An increasing body of evidence suggests that through the analysis of gene expression patterns of various cancer types, diagnosis, prognostic markers and new therapeutic targets can be identified^3^. Ovarian serous cystadenocarcinoma (OC) is the main type of ovarian cancer. Although a large number of studies have focused on the alterations of gene expression to explore OC, the underlying molecular mechanism associated with tumorigenesis and progression and their effects on the immune microenvironment remain largely unexplored.

Alternative splicing (AS) events constitute a prevalent mechanism in expanding the genetic diversity of eukaryotic cells. Spatiotemporal expression profiles of AS transcripts substantially contribute to cell differentiation, specification, and organogenesis^4^. In 1977, Phillip Sharp and Richard Roberts discovered the concept of “split genes” almost at the same time. Since then, AS events have been found to play important roles in many human diseases, including cancer. Several studies have identified that AS events were associated with the occurrence, development, and metastasis of multiple types of cancers^5–9^. A new study on AS events in ovarian cancer demonstrated that AS events can act as an independent prognostic signature for predicting ovarian cancer patients' survival outcome^10^. These findings suggest that AS events are valuable targets for cancer diagnosis, treatment, and prognosis prediction. Moreover, in order to obtain robust detection of disease-related alternative splicing events from the RNAseq data, Halperin et al. developed a new splicing data analysis package called Bisbee^11^. AS events have been reported closely related to tumor microenvironment (TME) which is a very complex system. TME is composed of tumor cells, immune and inflammatory cells, tumor-related fibroblasts, stromal tissues, and various cytokines and chemokines^12–15^. AS events is ubiquitous: 60% of genes showed frequent AS isoforms in T or B lymphocytes^16^. AS-influenced immune processes have captured great attention. With the development of next-generation sequencing technologies and the construction of The Cancer Genome Atlas (TCGA) database, the integrative analysis of RNA-sequencing data and prognosis information of patients makes it possible to systematically analyze the survival-related AS events in several types of cancers^17^. The prognostic values of AS events have been reported in patients with glioblastoma, breast cancer, lung cancer, and so on^18^. The correlation between survival-associated AS events and the immune microenvironment have also been confirmed in endometrial cancer, stomach adenocarcinoma, pancreatic cancer and so on^12,19,20^. Furthermore, dysregulated splicing factors (SFs) have been reported to cause global alteration of AS events in cancer^3^. In OC, a few studies have reported AS events by comparing cancer tissues with normal tissues^1,10,21,22^. However, the comprehensive pattern of AS events and the relationship between survival-associated AS events and immune microenvironment in OC has not been elucidated. With the development of tumor immunotherapies, two antibodies that targets the T cell checkpoint protein CTLA-4 and PD-1 have shown remarkable clinical effects^23^. However, there are few studies on treatment efficiency of CTLA-4 and PD-1 blockers in OC.

In the present study, we aimed to analyze the prognosis of AS events in OC through bioinformatics analysis, and explore the potential relationship between risk scores of patients and immune cells regulating OC comprehensively. We conducted systematic profiling of genome-wide AS events in patients with OC, which included 544 OC cases that were shared in TCGA SpliceSeq database and RNA-seq expression spectra. Moreover, we studied the relationship between aberrant AS events and the prognosis of patients with OC. Then Univariate and Multivariate Cox regression analyses were used to identify survival-associated AS events and build a prognostic model based on 11-AS events. The Kaplan–Meier (K–M) curve and receiver operating characteristic (ROC) curve were performed to demonstrate the prognostic value of the prognostic model. Furthermore, we used the clinical data from TCGA database to explore the relationship between AS events and clinical features. Besides, the distribution of immune cells in OC between the high- and low-risk groups were displayed. We also conducted K-M survival curves to explore the immune cells associated with prognosis. The immunotherapy score (IPS) of CTLA-4 and PD-1 blockers in OC were downloaded from The Cancer Imaging Archive (TCIA) database. We compared the immunotherapeutic effect of CTLA-4 and PD-1 blockers in the two subgroups. In addition, SFs associated with AS events were also identified, and the relationship network between AS events and SFs was established. The AS-SF correlation network revealed several hub SF genes, including DDX39B, PNN, LUC7L3, ZC3H4, and SRSF11. Collectively, our findings shed new light on developing immune targeted therapy and improving the prognosis of patients with OC.

## Materials and methods

### Data collection and processing

The RNA transcriptome profiles and clinical information of 550 patients with OC were retrieved from TCGA database^24^. Patients with a follow-up of fewer than 90 days were excluded because these patients might die due to other factors, such as surgical complications. At the same time, we retrieved the Percent-spliced-in (PSI) data more than 75% of AS events from TCGA SpliceSeq database and the number of samples is 412. A total of 384 patients were finally eligible. PSI values ranging from 0 to 1 were used to quantify the AS events^25^. Subsequently, the AS events were annotated by combining the splicing type, ID number in the SpliceSeq, and the corresponding parent gene symbol^26^. Seven types of AS events were included in the present study, such as exon skipping (ES), mutually exclusive exon (ME), retained intron (RI), alternate promoter (AP), alternate terminator (AT), alternative donor site (AD), and alternative acceptor site (AA).

### Survival-associated AS events in OC

Firstly, we analyzed the distribution of all genes in seven different types of AS events in OC. Different AS events of gene occurrence led to the diversity of results, and different gene expression led to the change of survival time. The PSI of AS events in patients with OC was supplemented by Knn function in the R language Impute package, which was then integrated with patient survival time and survival status. Univariate Cox regression was used to evaluate the correlation between PSI value and overall survival of patients with OC and screen the survival-associated AS events with a *P*-value < 0.05^27^. Upset plots were created by UpSet R to visualize the intersections of all seven types of survival-related AS events in OC. Besides, the bubble charts were used to summarize the top 20 AS events of each type, except for the ME events, which only had eight survival-related AS events.

### Construction of the prognostic model for OC

Lasso regression analysis was employed to select survival-associated AS events in each splicing type, which could avoid over-fitting. Then the prognostic model based on 11-AS events was constructed by multivariate Cox analysis. The risk score of the prognostic model was calculated for the prediction of OC, and the formula used for calculating the risk score for each patient was as follows: Riskscore = β_AS event1_ × PSI_AS event1_ + β_AS event2_ × PSI_AS event2_ + · · · + β_AS eventn_ × PSI_AS eventn_. Then the patients were divided into two subgroups (high-risk and low-risk) according to the median risk score. There were 192 cases in each subgroup. K-M survival curves with Log-Rank test was performed to compare the overall survival effect of the prognostic model in two risky sets. The time dependent ROC curve was performed by the “survival ROC” R package and the area under the curve (AUC) was calculated to assess the predictive power of the prognostic model. The detailed information of AS events, including the distribution of risk score, the distribution of survival time, and the expression heatmap, were also visualized.

### Estimation of independent prognostic value

The risk score of the prognostic model and two important clinical features, including grade and age, were integrated into the univariate and multivariate Cox regression analyses to evaluate whether these features could be used as independent risk factors.

### Validation the survival correlation between prognostic model and immune cells in patients with OC

Single-sample Gene Set Enrichment Analysis (ssGSEA) was used to calculate immune score to predict the level of immune cells in OC tissues^28^. Then we screened the immune cells related to survival-associated AS events. A total number of 23 immune cells and 259 patients were included for further analysis. Patients were separated into two (high/low) risk groups by the median value based on the risk score calculated by prognostic model. The R package was used to compare the distribution of immune cells between the two groups. Then we chose activated B cell for Pearson correlation analysis with other 6 kinds of cells which showed significant differences between the two groups using Graphad prism 8. K-M survival curves for survival-related immune cells were also performed in OC.

### IPS predicts response to immunotherapy with CTLA-4 and PD-1 Blockers in OC

The IPS of CTLA-4 and PD-1 blockers in 260 patients with OC were downloaded from the TCIA database. These 260 patients also were classified into low- and high-risk groups according to risk score of the prognostic model. Then we compared the distribution of IPS of CTLA-4 and PD-1 blockers between the two groups using R package. A violin diagram was used to visualize the different treatments response between two groups.

### Potential correlation network of survival-associated AS and SFs

SFs can regulate AS events by binding to pre-mRNAs, affecting exon selection and choice of splicing site^29^. To analyze the correlation between survival-associated AS events and SFs, a regulatory network was constructed between SF genes and AS events. The expression data of SFs were extracted from TCGA database. The correlation between the SFs and these survival-associated AS events was analyzed using Pearson’s correlation test. The AS-SF correlation network was plotted and visualized using the Cytoscape (3.7.1) software.

### Functional enrichment analysis

To further explore the underlying mechanisms of AS in OC, we identified corresponding SF genes of AS events. Functional enrichment analysis was carried out using the Database for Annotation, Visualization, and Integrated Discovery (DAVID) online functional annotation tool. Gene ontology (GO) terms and Kyoto Encyclopedia of Genes and Genomes **(**KEGG) pathways with *P* < 0.05 were considered a statistically significant difference. KEGG includes most known metabolic pathways and some known regulatory pathways. It can be used to reveal the molecular interactions and pathways behind the gene expression profile obtained by microarray experiment^30,31^. The SFs related to survival-associated AS events were selected as candidates for GO and KEGG pathway enrichment analysis. Both GO analysis and KEGG analysis were conducted using R × 64 3.6.1 software.

### Ethical approval

Ethical approval was not necessary because this work is a bioinformatics analysis. All the data were downloaded from the online databases. All the methods in the study were carried out in accordance with relevant guidelines and regulations.

## Results

### Details of AS events

**Figure 1:**
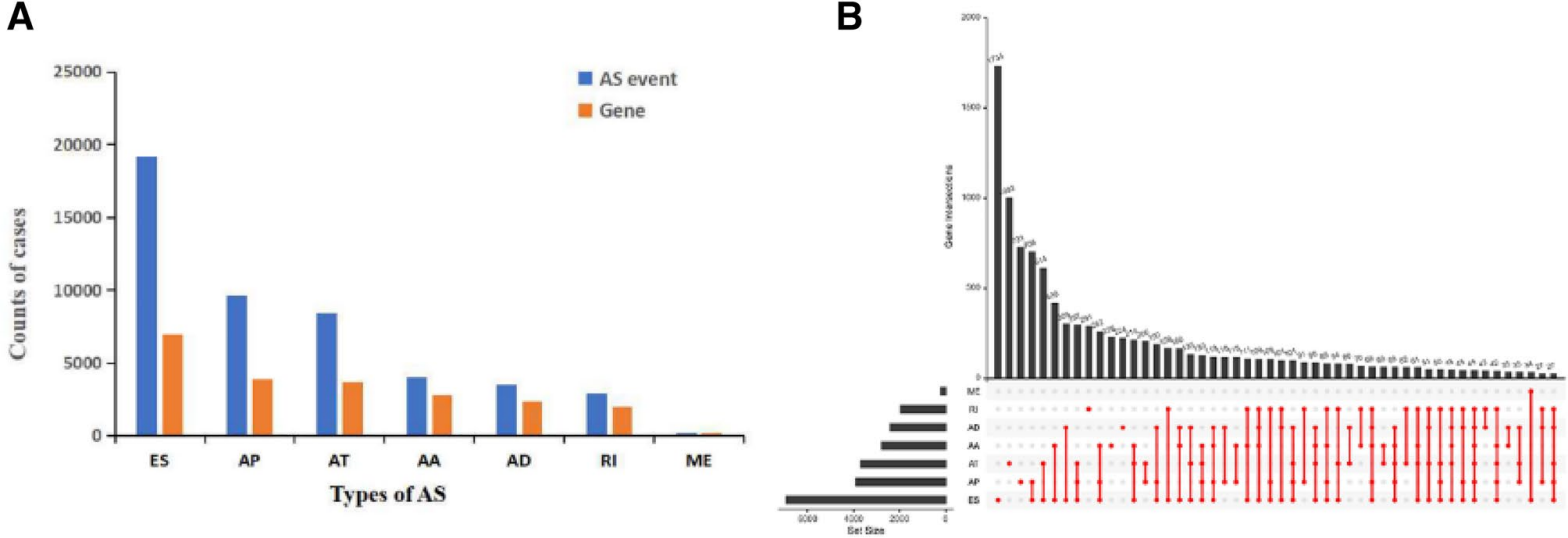
Summary of AS events of OC. (**A**) Counts of AS events and correlated genes. (**B**) Upset plot in OC, showing the interactions among seven types of AS events. One gene may have up to five types of AS events.

A total of 544 patients with OC were included in the present study and the baseline characteristics of patients are summarized in Table 1. Totally 47,922 AS events in 21,794 gene symbols were identified. The AS events consisted of 3995 AAs in 2770 genes, 3494 ADs in 2386 genes, 9652 APs in 3889 genes, 8438 ATs in 3685 genes, 19,197 ESs in 6916 genes, 207 MEs in 201 genes, and 2939 RIs in 1947 genes (Fig. 1A). The results showed that ES was the main splicing pattern, while ME was the least frequent event among the seven types of AS events in OC. It was important to note that the number of AS events far exceeded the number of genes. Figure 1B shows that one gene could undergo up to five types of AS events.

**Table 1:** Clinical parameters of ovarian cancer patients from the TCGA.

Parameter/feature	No. of patients (%)
	(n = 544)
**Age**	
Median	59 (26–87)
< 60	291 (53.5%)
≥ 60	253 (46.5%)
**Sex**	
Male	0 (0%)
Female	544 (100%)
**Tumor grade**	
G1	6 (1.1%)
G2	66 (12.1%)
G3	459 (84.4%)
G4	1 (0.2%)
GB	1 (0.2%)
GX	9 (1.6%)
Unknown	2 (0.4%)

### Survival-associated AS events

**Figure 2:**
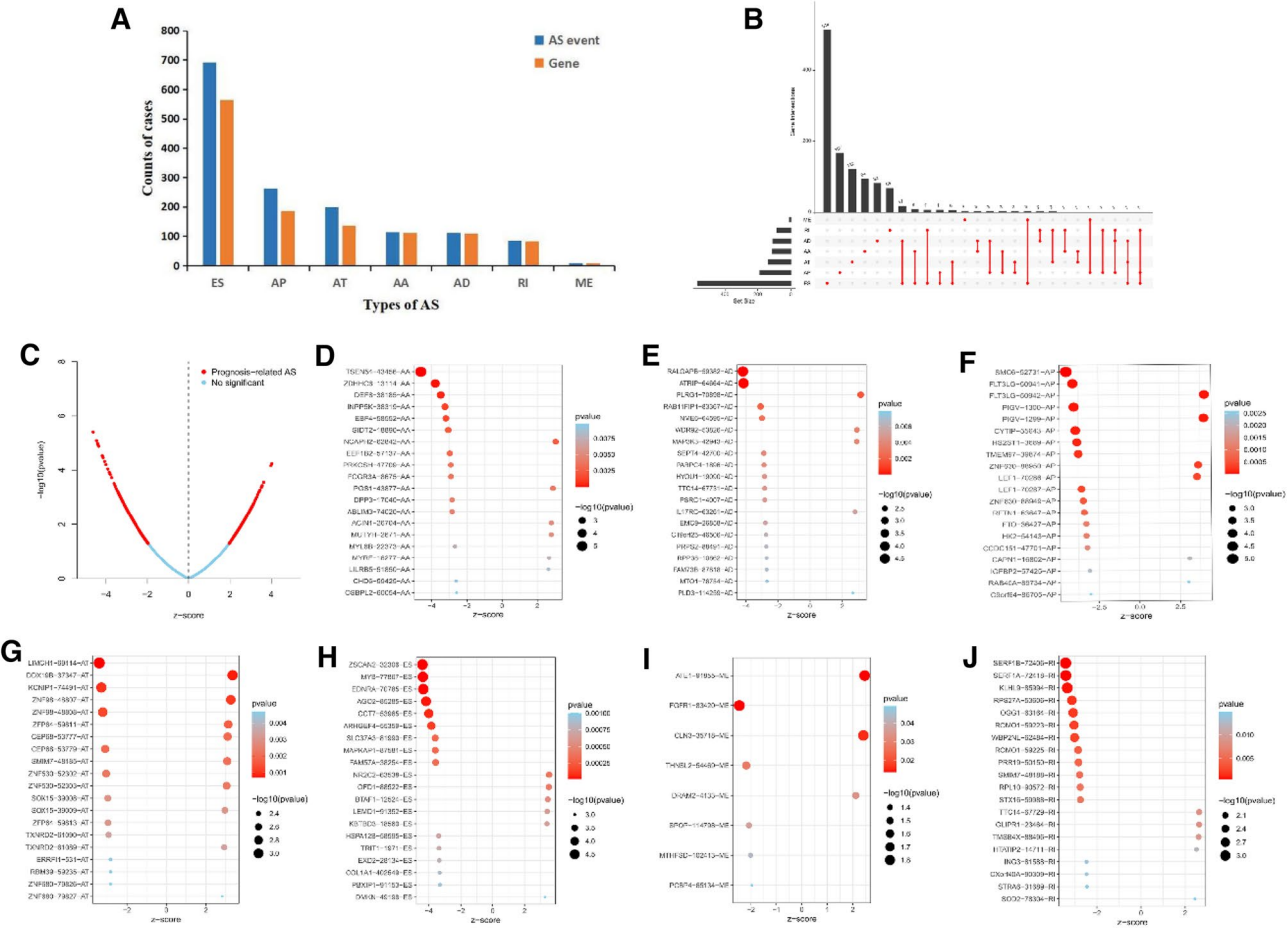
Top 20 significant AS events of OC. (**A**) Counts of survival-associated AS events and correlated genes. (**B**) Upset plot of interactions diagram of survival-associated AS events. (**C**) The volcano plot of survival-related AS events (red dots). Red dots indicate survival-related AS events in OC. Blue dots indicate AS events unrelated to survival in OC. Bubble plots of the top 20 survival-related AS events based on AA (**D**), AD (**E**), AP (**F**), AT (**G**), ES (**H**), ME (**I**), and RI (**J**), respectively.

We identified 1472 AS events using the AS event profiles in the OC cohort, which were significantly associated with overall survival (OS) of patients with OC by univariate Cox regression analysis (*P* < 0.05). Figure 2A lists the number of each type of AS event. To better visualize the intersection, an UpSet plot was created as shown in Fig. 2B, and we found that up to three survival-associated AS events could occur in the same gene. Specifically, ES, AP, AT, AA, AD, and RI were all significantly linked to the OS of patients. Figure 2C indicates the AS events that were associated with survival of patients (red dots) and not associated with survival of patients (blue dots), showing that most AS events were significantly associated with patients’ survival. Figure 2D-J showed the top 20 most significant survival-associated AS events of each type. For ME events, only eight AS events were related to survival.

### Prognostic model selection and survival analysis

**Figure 3:**
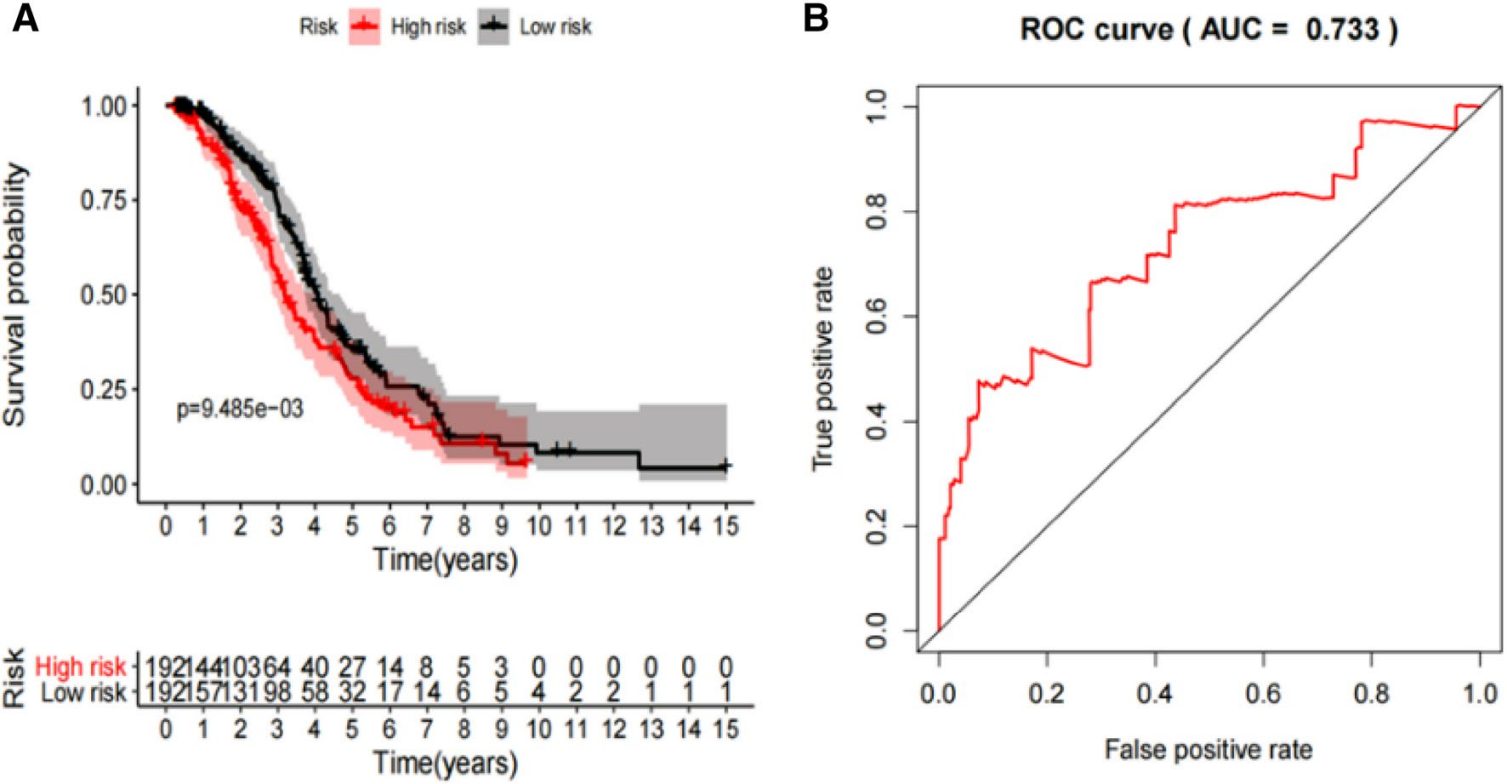
The K-M curve indicated the OS of high-risk patients (red line) and low-risk patients (blue line) based on 11-AS events (**A**). The ROC curve evaluated the predictive power of prognostic model and the risk score reflected the greatest prognostic power with an AUC of 0.733 (**B**).

Lasso regression analysis was performed to avoid over-fitting and exclude the co-expressed AS events, which were selected by Univariate Cox analysis. Supplementary Fig. 1 presents the result of Lasso regression analysis of survival associated AS events, and we selected the most highly correlated AS events. Multivariate Cox analysis was then used to construct predictive model and calculate the risk score. Table 2 lists the prognostic model of 11-AS events. Patients were then divided into high-risk and low-risk subgroups according to the median risk score of the prognostic model. There were 192 cases in each subgroup and the median risk score was 0.9137. According to K-M survival analysis, we found that the prognostic model played significant roles in distinguishing good or poor outcomes of patients (Fig. 3A). We plotted the ROC curve and calculated the AUC to verify the efficiency of the predictor. The result revealed that the AUC of 11-AS events was 0.733 (Fig. 3B). Supplementary Fig. 2 illustrated the distribution of patients’ survival status (A), risk score (B), and the expression heatmap (C) of the prognostic model. The risk curve showed the result of patients ranking based on the risk score. There was a difference between the high-risk group and the low-risk group in risk score. The survival status of patients disclosed that there were higher mortality rates in the high-risk group (green dots represent survival, and red dots represent death). The color transition from green to red in the heat map indicated that the PSI score of the AS events was increased from low to high.

**Table 2:** Mutivariate Cox analysis of prognostic AS predicting overall survival.

Gene symbol	Spliceseq ID	AS type	HR.95L	HR.95H	pvalue
TSEN54	43,456	AA	5.29E-20	2.25E-06	0.000337526
SMC6	52,731	AP	3.31E-05	0.244173502	0.0098632
EDNRA	70,785	ES	0.000555224	0.243945187	0.00412093
AGO2	85,285	ES	4.80E-08	0.001887305	1.83E-05
ATRIP	64,664	AD	0.000520359	0.103314245	0.000270829
FLT3LG	50,941	AP	0.037905348	0.524319914	0.00346249
CCT7	53,965	ES	0.023328416	0.33048535	0.000321669
PIGV	1300	AP	0.09617422	0.619578962	0.003004361
CYTIP	55,643	AP	4.50E-07	0.012016318	0.000251836
ZDHHC6	13,114	AA	0.00016264	0.097870088	0.00071571
ZNF630	88,950	AP	2.328594437	80.61439575	0.003794231

### Estimation of independent prognostic value

**Figure 4:**
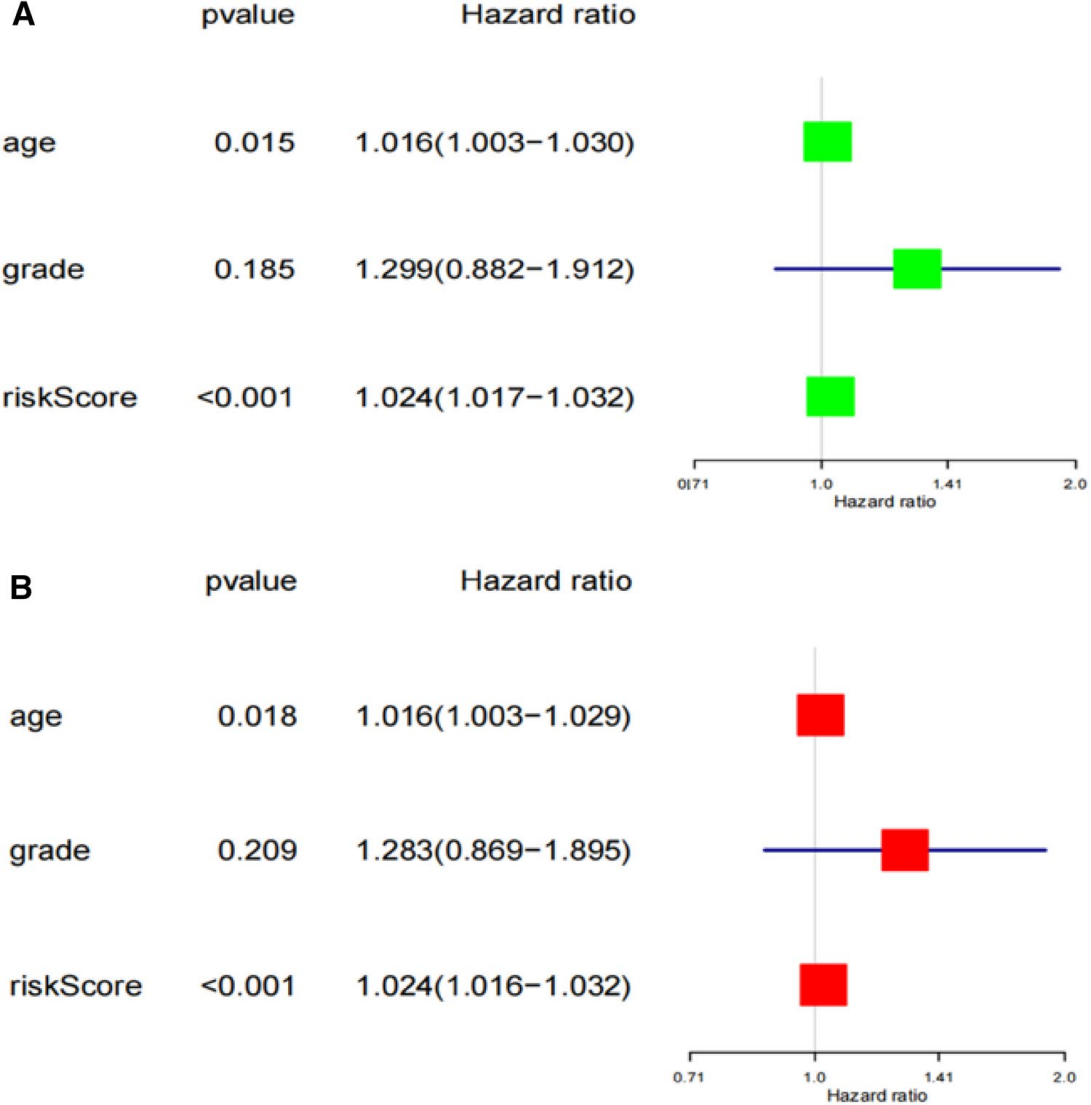
The prognostic value of age, stage, and risk score in OC. Univariate (**A**) and Multivariate Cox (**B**) regression analyses of the prognostic model.

We used Univariate and Multivariate Cox regression analyses to estimate the independent prognostic value of age, grade, and risk score of the prognostic model. Univariate Cox regression and Multivariate Cox regression analysis indicated that both age and risk score could predict survival of OC and were independent prognostic predictor (Fig. 4).

### The tumor immune microenvironment was closely related to the prognosis of OC

**Figure 5:**
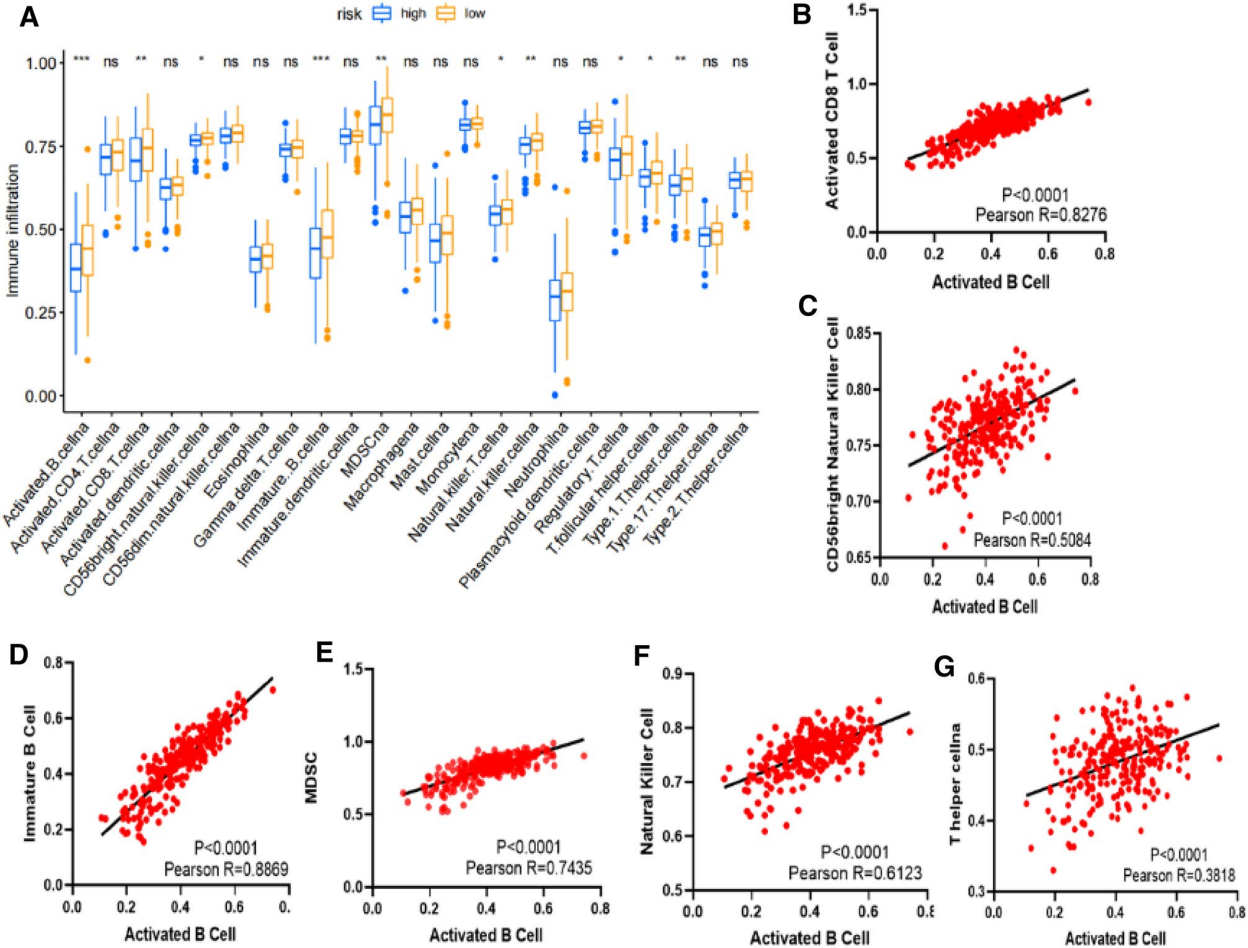
The riskscore is associated with the immune cells infiltrating in OC microenvironment. (**A**) Differences in the infiltrating proportion of 23 types of immune cells in two groups (low: 132 patients, high: 127 patients). (**B**) The correlation analysis showed that activated B cell was positively correlated with activated CD8 T cell, CD56 bright natural killer cell, immature B cell, MDSC, natural killer cell and type 1 T helper cell.

**Figure 6:**
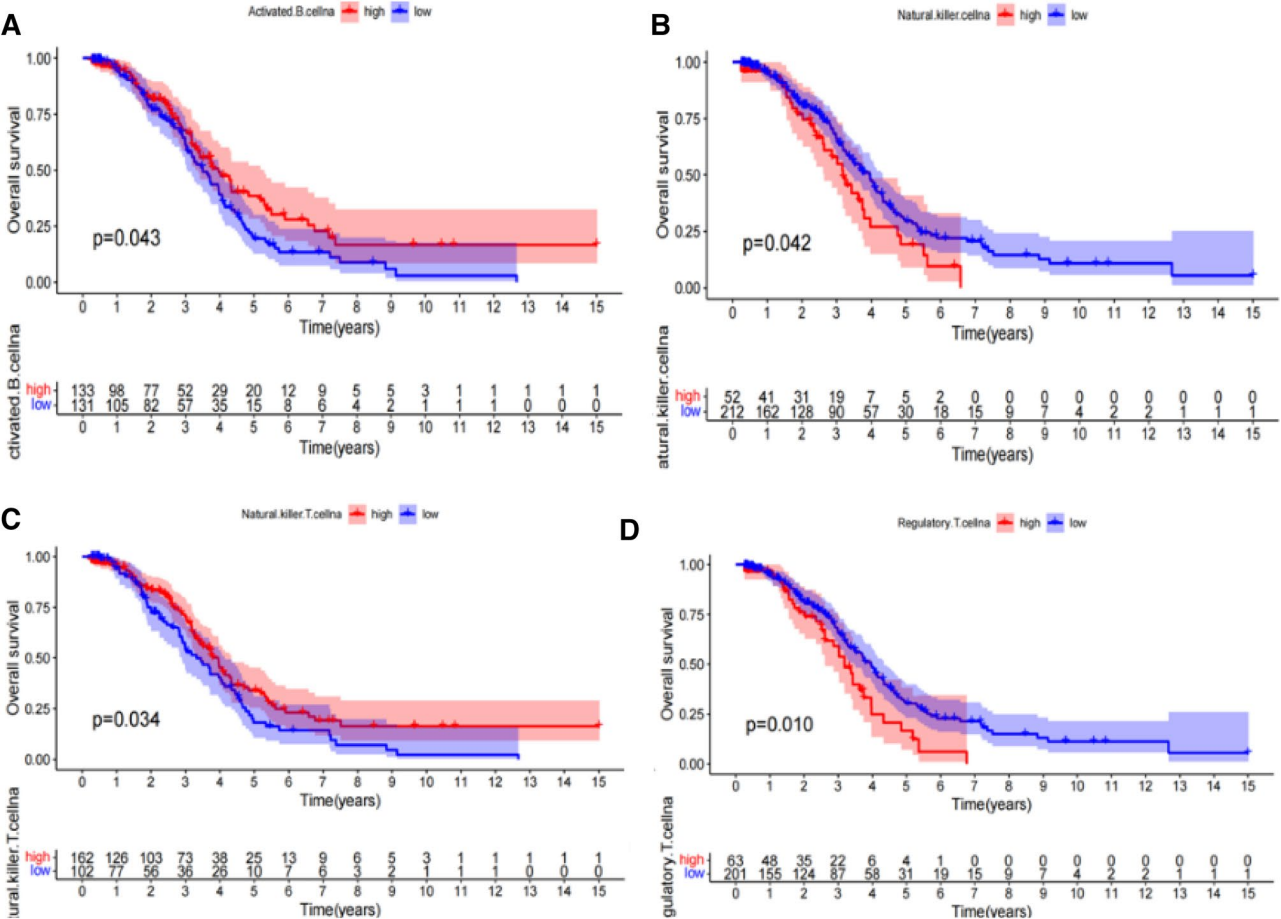
Kaplan–Meier survival curve of patients with survival-related immune cells. High expression of activated B cell (**A**) and natural killer T cell (**C**) was beneficial to the prognosis of patients, while natural killer cell (**B**) and regulatory T cell (**D**) was on the contrary.

We divided 259 patients into two groups based on the risk score calculated by prognostic model (low: 132 patients, high: 127 patients). Then the immune infiltration of the 23 immune cells were compared in these two subgroups. The proportion of 10 immune cells was significantly higher in low-risk group, including activated B cell, activated CD8 T cell, CD56 bright natural killer cell, immature B cell, MDSC, natural killer T cell, natural killer cell, regulatory T cell, T follicular helper cell and type 1 T helper cell (Fig. 5A). The correlation analysis showed that activated B cell was positively correlated with activated CD8 T cell, CD56 bright natural killer cell, immature B cell, MDSC, natural killer cell and type 1 T helper cell (Fig. 5B-G). Then we conducted K-M survival curves on these 10 kinds of immune cells and found four types of cells, including activated B cell, natural killer cell, natural killer T cell and regulatory T cell, which were associated with prognosis of OC (Fig. 6). We also found that high expression of activated B cell and natural killer T cell was beneficial to the prognosis of patients, while natural killer cell and regulatory T cell was on the contrary.

### The response of immunotherapy with CTLA-4 and PD-1 Blockers in OC

**Figure 7:**
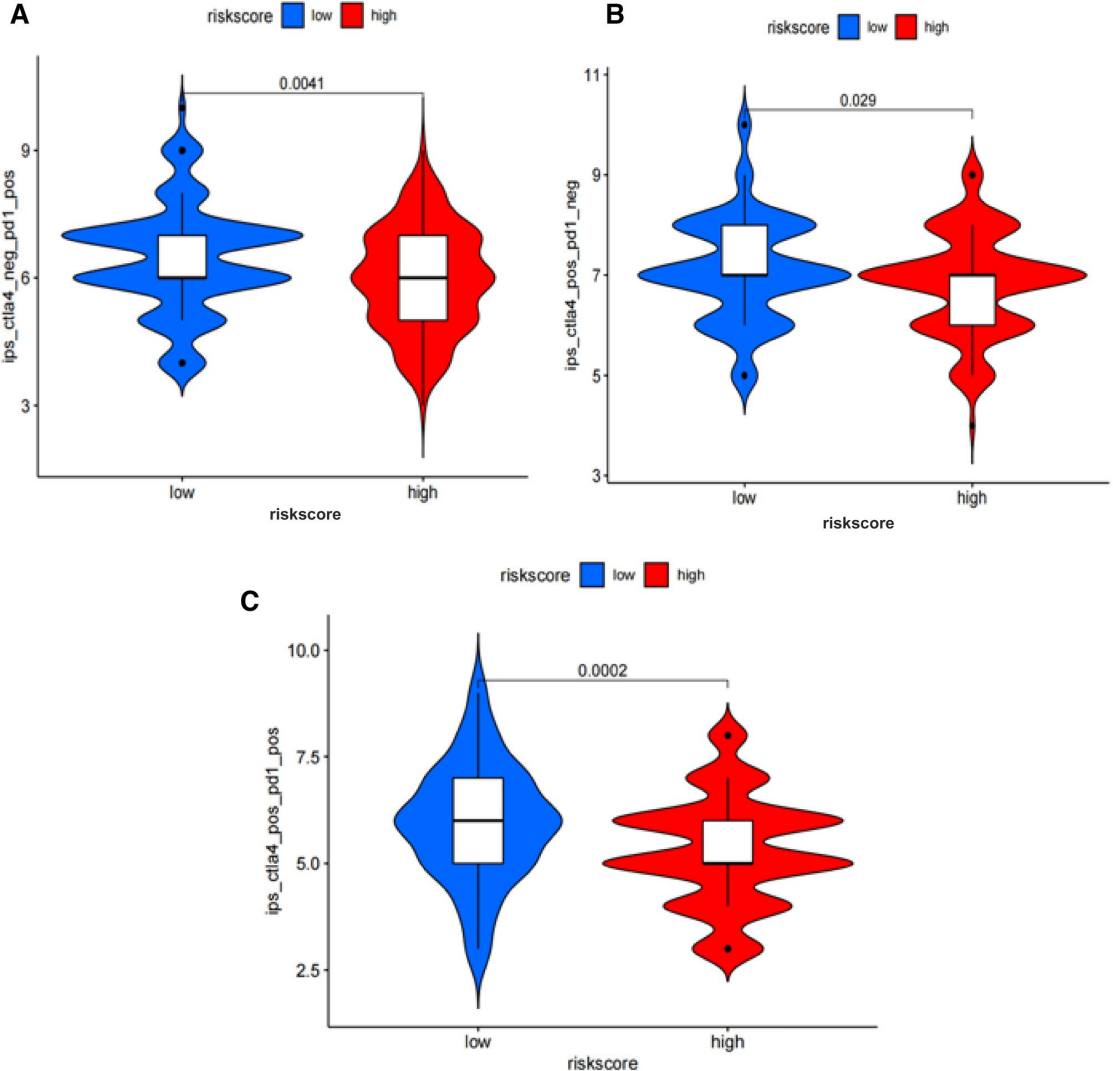
Violin diagram was used to visualize the different treatments (CTLA-4) response between low- and high- risk patients. The patients in low-risk group had good response to the single (**A**, **B**) and combined (**C**) use of the two drugs.

We compared the IPS of CTLA-4 and PD-1 blockers in patients with OC of high and low risk groups. The results indicated that the patients in low-risk group had good response to the single and combined use of the two drugs (Fig. 7).

### Correlation network of SFs

**Figure 8:**
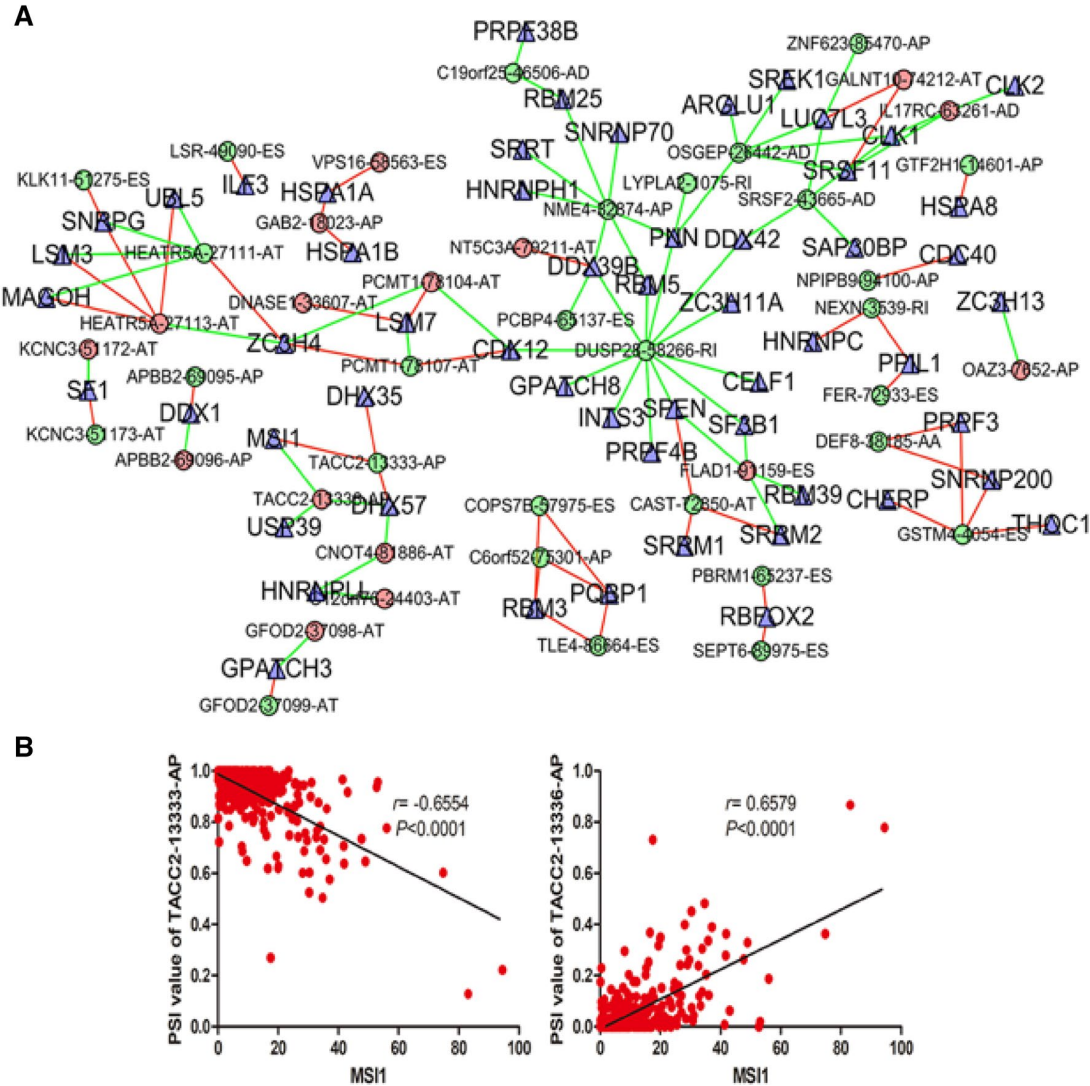
The interaction network and correlation between SFs and AS events. (**A**) The red dots indicate down-regulation, and the green dots indicate up-regulation. The blue triangles indicate SFs. The green lines represent AS events, which were positively correlated with the expressions of SFs, while red lines indicate negative correlations. (**B**) The correlation analysis was performed using Pearson *t* test. MSI1 could positively regulate -13336-AP (*r* = 0.6579, *P* < 0.0001) and negatively regulate TACC2TACC2-13333-AP (*r* = − 0.6554, *P* < 0.0001).

To analyze the correlation between survival-associated AS events and SFs, an AS-SF network was constructed based on the result of Pearson’s correlation test. Figure 8A showed that the network contained 56 SFs (blue triangles) and 104 survival-associated AS events, including 45 down-regulated AS events and 59 up-regulated AS events (red and green dots). The green lines represented AS events, which were positively correlated with the expressions of SFs, while red lines indicated negative correlations.

Interestingly, we found that MSI1 could positively regulate TACC2-13336-AP and negatively regulate TACC2-13333-AP. A total of 359 OC tumor tissues were used to show the correlation between expression of MSI1 and PSI value of TACC2-13336-AP (*r* = 0.6579, *P* < 0.0001), TACC2-13333-AP (*r* = − 0.6554, *P* < 0.0001) (Fig. 8B).

### GO functional and KEGG pathway enrichment analyses of OC

**Figure 9:**
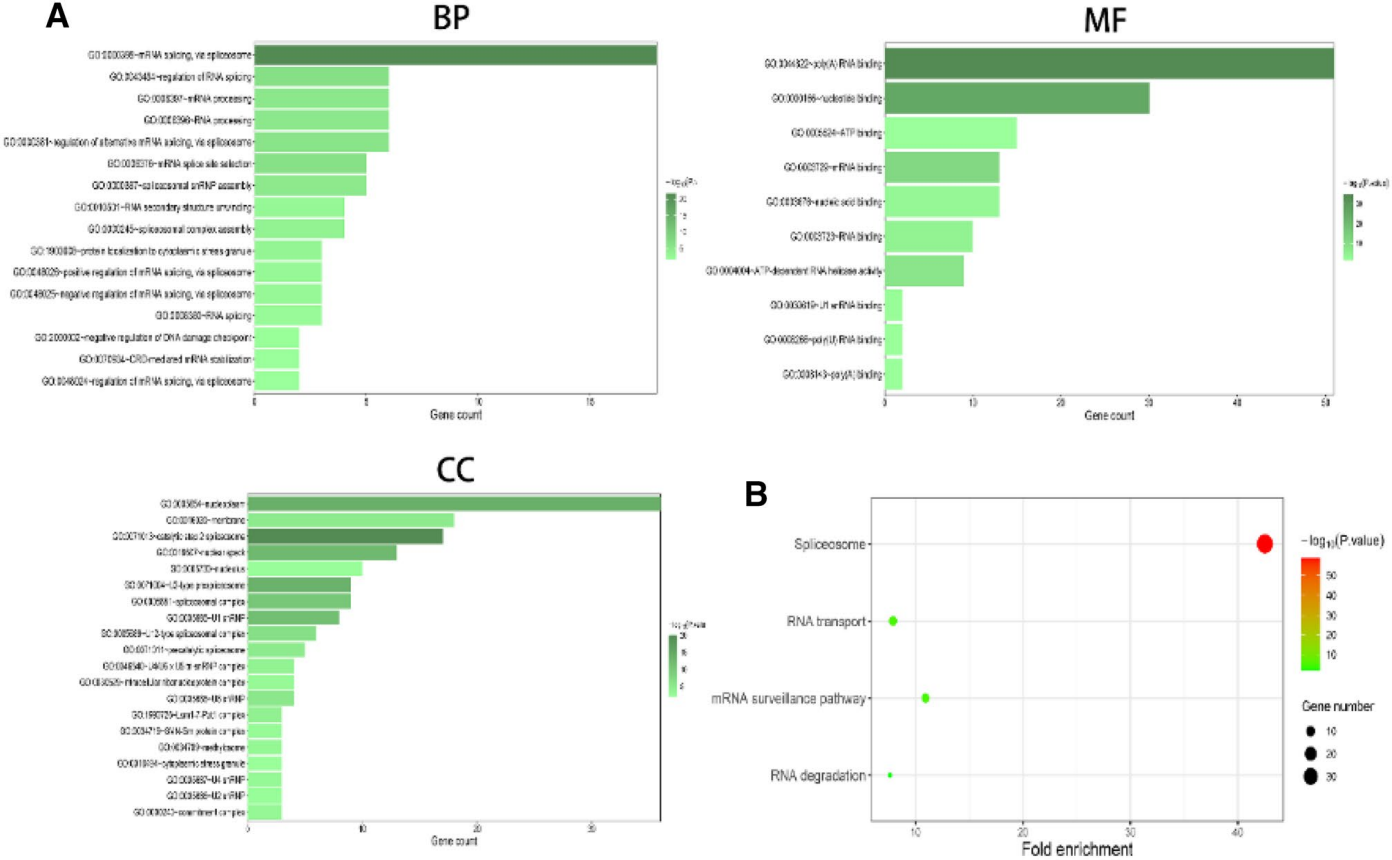
GO functional enrichment analysis (**A**) and KEGG pathway analysis (**B**) of AS event-related SF genes.

GO analysis demonstrated that “mRNA splicing via spliceosome”, “regulation of RNA splicing”, “mRNA processing”, “RNA processing”, and “regulation of alternative mRNA splicing via splicesome” were the most significant biological process terms. Moreover, “nucleoplasm”, “membrane”, “catalytic step 2 splicesome”, and “nuclear speck” were the most three significant cellular component terms. Besides, “poly (A) RNA binding”, “nucleotide binding”, and “ATP binding” were the most three significant molecular function terms (Fig. 9A). KEGG analysis revealed four remarkably enriched pathways, including “spliceosome”, “RNA transport”, “mRNA surveillance pathway”, and “RNA degradation”. It also revealed that these genes were mainly involved in the “spliceosome” pathways (Fig. 9B).

## Discussion

AS event is one of the main engines driving proteome diversity. It is estimated that up to 94% of genes are alternatively spliced in humans. Just as many other cellular processes are modified during cellular growth, differentiation, and tissue development, AS events are also affected^32^. AS allows cells to generate diverse mRNA by modifying mRNA isoforms. The plasticity of AS is often exploited by cancer cells to produce isoform switches that promote cancer cell survival, proliferation, metastasis, and drug resistance. In recent years, it has been proved that AS events play an important role in the occurrence and development of several types of tumors. For example, AS event of TCF‐4 is found to inhibit the proliferation and metastasis of lung cancer cells^33^.TP53, FAS, CASP9, and BCL2L1 are also associated with the apoptosis and survival of cancer cells^34^. Calabretta's study has revealed that modulation of the pyruvate kinase gene (PKM) splicing can promote gemcitabine resistance in pancreatic cancer cells^35^. Recently, with the development of bioinformatics technology, TCGA project contains a large amount of RNA-seq data, PSI value of AS events, and clinical information of patients, which provides a rich source for the exploration of the relationship between AS events and the prognosis of cancer patients^33–35^. Associations between AS events and prognosis of patients have been demonstrated in non-small cell lung cancer (NSCLC), adrenocortical carcinoma (AC), head and neck squamous cell carcinoma (HNSCC), and so on. Previous studies have demonstrated the role of several AS patterns in OC. Dutta et al. have reported that EVI1 is frequently aberrantly spliced in OC, and the dominant form of EVI1 (EVI1^Del190–515^) plays oncogenic roles in the tumorigenesis of OC^36^. Sosulski et al. have reported that CD44 variants containing exons v8–10 (CD44 v8–10) are associated with metastasis and worse prognosis in OC^37^. Although these reports provide evidence for the involvement of AS events in OC, it is still urgently necessary to systematically analyze the characteristics of AS events, which may provide potential prognostic biomarkers and therapeutic targets.

To explore the prognostic significance of AS events, we identified survival related AS events and constructed predictive model for OC. In the present study, we systematically described the AS profiles and explored the interaction network between AS events and SFs in OC. A total of 47,922 AS events of 21,794 genes were detected, indicating that AS was a common process in OC. ES events were the main type, accounting for 1/3 of the total AS events. AP events were the second most frequently occurring type, followed by AT events. Next, Lasso regression and Multivariate Cox regression analysis were performed to construct the prognostic model. These patients with OC from TCGA database were then divided into low-risk and high-risk groups according to the risk score. K-M analysis demonstrated that the difference in OS between the low-risk patients and high-risk patients was significant. The results revealed that the risk score of the prognostic model consisting of 11 survival-related AS events had a prognostic power with an AUC of 0.733.

Immune cells in tumor microenvironment are also accompanied by the tumorigenesis and progression of cancer. More and more studies have reported that the products of AS events in cancer cells were also affected the immune system^28,38^. So AS events also have shown potential immunotherapy prospects, but how AS events affected the immune system of OC and whether AS events could be a target for diagnosis and therapy remain unclear. Hoyos, L. E.’s study has shown that T lymphocytes recognized neoepitopes derived from abnormal AS events to induce antitumor immune response^39^. Thus, we analyzed the association between the risk score based on 11 survival-related AS events and 23 immune cells in the immune microenvironment. The result showed that a low-risk score was significantly associated with upregulated activated B cell, activated CD8 T cell, CD56bright natural killer cell, immature B cell, MDSC, natural killer T cell, natural killer cell, regulatory T cell, T follicular helper cell and type 1 T helper cell. Activated B cell, natural killer T cell, natural killer cell and regulatory T cell. We also found there were correlations between these immune cells through correlation analysis. Activated B cell, activated CD8 T cell, natural killer cell, natural killer T cell and regulatory T cell were also associated with prognosis of OC according to K-M survival curves. Several previous immunotherapy studies have demonstrated that efficiency of CTLA-4 and PD-1 blockers has been shown not only in melanoma, but also in nine different tumor types^40,41^. We downloaded the IPS of CTLA-4 and PD-1 blockers in 260 patients from the TCIA database. The result showed that the patients in low-risk group had better response to the CTLA-4 and PD-1 blockers. Moreover, studies have shown that neo-vessel formation was regulated by the interaction between endothelial cells and the microenvironment around vessels. Especially in the inflammatory environment, immune cells are the key cells for the formation of new vessels, including neutrophils, macrophages and lymphocytes around vessels^42^. Tumor angiogenesis has always been found to be one of the key features of cancer. Solimando, A. G.’s research has also shown that the interference between adaptive immune cells and tumor endothelial cells is very important for the success of tumor immune monitoring and immunotherapy using immune cells to kill tumor cells^43^. These findings also inspire us to further explore the interaction between immune cell infiltration and angiogenesis in ovarian cancer, thus providing new ideas for the mechanism and treatment of ovarian cancer.

An interaction network between AS events and SFs was also established. The network contained 56 SFs and 104 survival-associated AS events (including 59 up-regulated AS events and 45 down-regulated AS events). Previous studies have shown that a single gene could regulate multiple AS events of the same parental gene, even in opposite way. Interestingly, our results indicated that MSI1 could positively regulate TACC2-13336-AP and negatively regulate TACC2-13333-AP which was consistent with previous reports.

Besides, GO functional enrichment and KEGG pathway analysis for the SFs significantly related to AS events provided helpful clues to elucidate the underlying mechanism of AS events in OC. According to the results, “mRNA splicing via spliceosome”, “regulation of RNA splicing”, “mRNA processing”, “RNA processing”, and “regulation of alternative mRNA splicing via splicesome” were the most significant biological process terms. Moreover, “nucleoplasm”, “membrane”, and “catalytic step 2 splicesome” were the most three significant cellular component terms. Besides, “poly(A) RNA binding”, “nucleotide binding”, and “ATP binding” were the most three significant molecular function terms. KEGG pathway analysis revealed that these genes were mainly involved in the “spliceosome” pathways.

Although our predictor performed well in prognosis prediction of OC, there were inevitably several limitations in the current study. First of all, the data we collected from public databases were limited. Therefore, the clinical information was not comprehensive and might cause potential bias and errors. Second, we explored prognosis-related AS events and established valuable prognosis signature of OC based on TCGA and SpliceSeq databases in this study which provide a rich source of AS events and corresponding detailed clinical information of patients. The integrative analysis of SplieceSeq data and clinical information of OC makes it possible to systematically analyze the survival-related AS events which overcomes the limited number of sequencing samples in a single laboratory and the difficulty of following up patients’ outcomes. Unfortunately, it is difficult for our team to acquire survival information of large portion of ovarian cancer patients, thus it is hard to finish validating the prognosis value of established signatures. Furthermore, our exploration of the mechanisms was not deep enough and further studies such as molecular and clinical trials, are necessary to confirm these findings. Our data revealed the prognostic value of survival‐associated AS events and related SFs, which might play essential roles in tumor initiation and progression by regulating the corresponding AS events. Collectively, our findings might provide valuable insights into effective therapies using AS events for OC.

### Data access

The RNA transcriptome profiles and clinical information of the OC cohorts were downloaded from the TCGA database. The IPS of CTLA-4 and PD-1 blockers in OC were downloaded from the TCIA database.

## Supplementary Information

Supplementary Information.
