# Supplementary material for: Comprehensive characterization of 11 prognostic alternative splicing events in ovarian cancer interacted with the immune microenvironment
Source: Sci Rep. 2022 Jan 19;12:980. doi: 10.1038/s41598-021-03836-1 (PMC8770494; doi:10.1038/s41598-021-03836-1)
Supplement: Supplementary file 1 — Supplementary Information. [file 41598_2021_3836_MOESM1_ESM.pdf]

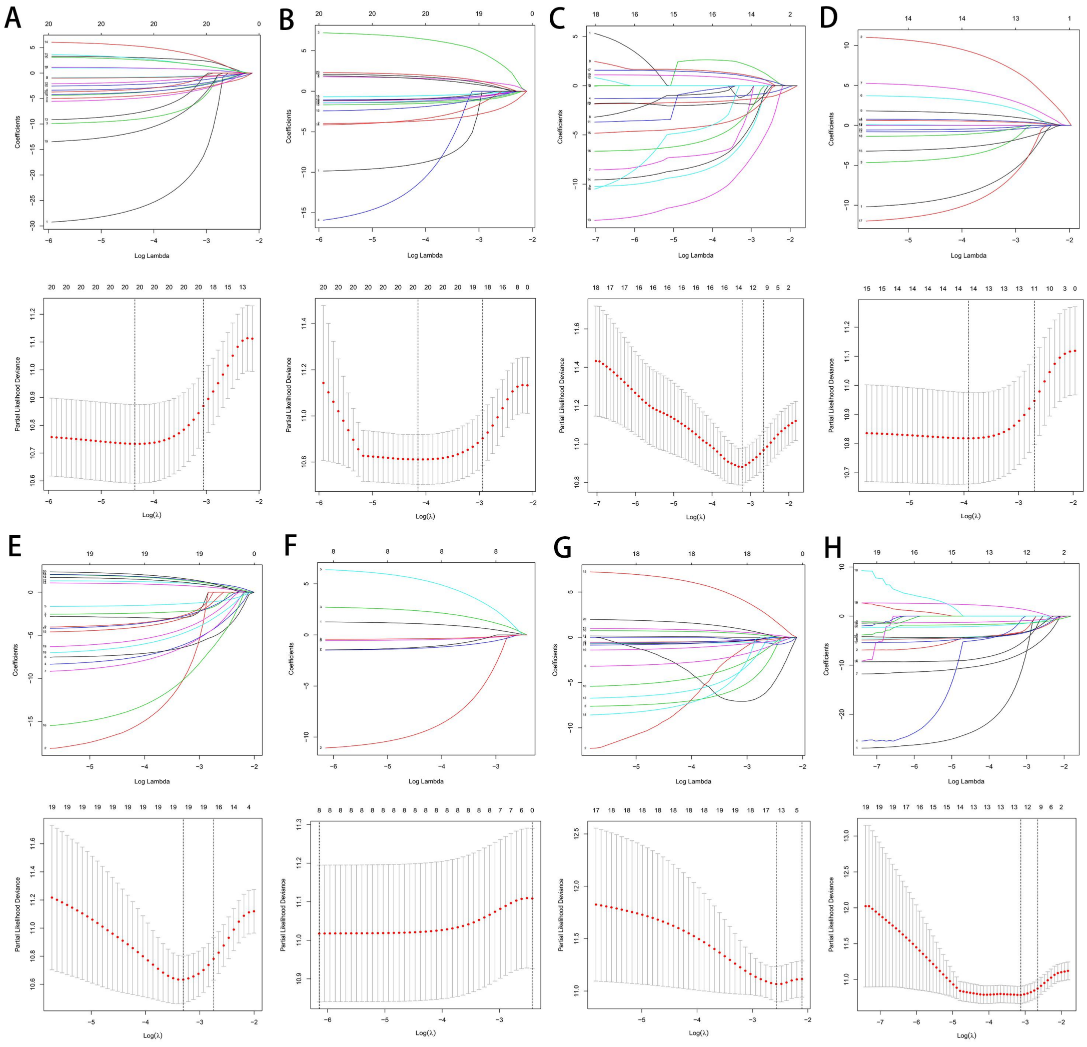

Supplementary Figure 1. Prognostic signatures of survival-related AS events constructed based on Lasso Cox analysis. AA cohort (A), AD cohort (B), AP cohort (C), AT cohort (D), ES cohort (E), ME cohort (F), RI cohort (G), and the whole cohort (H).

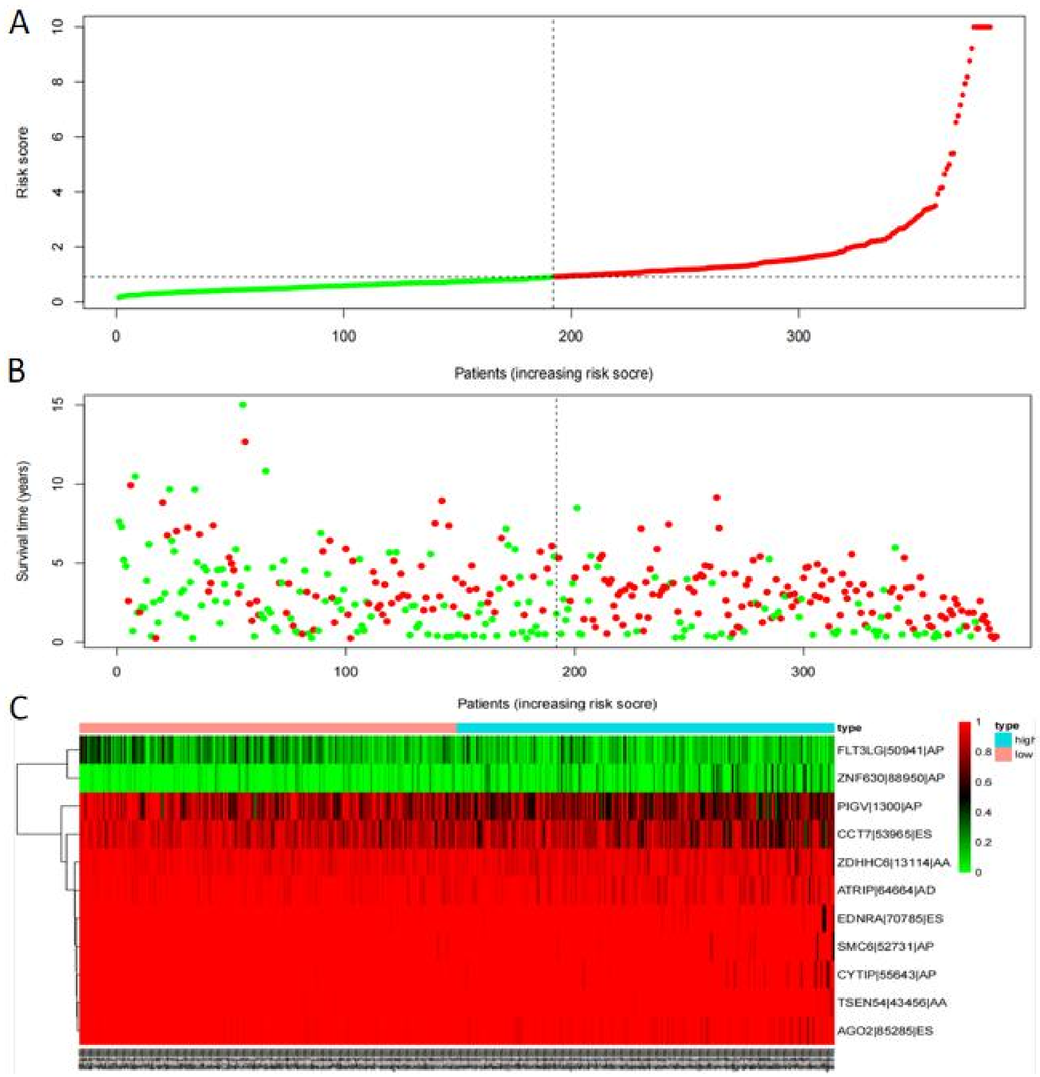

Supplementary Figure 2. Patients were then divided into high-risk and low-risk subgroups according to the median risk score of 11-AS events. The upper part is the risk score of each individual (A). The middle part is the survival status of each individual (green dots represent survival and red dots represent death) (B). The bottom part is the heatmap of AS events. The color transition from green to red indicates the increasing PSI score of corresponding AS events from low to high (C).
